# Reply to Trudeau, M.; Fraser, B. The CADTH pCODR Expert Review Committee Process Explained. Comment on “Rayson et al. Access to Neoadjuvant Pertuzumab for HER2 Positive Breast Cancer in Canada: A Dilemma Increasingly Difficult to Explain. *Curr. Oncol.* 2022, *29*, 9891–9895”

**DOI:** 10.3390/curroncol30050381

**Published:** 2023-05-16

**Authors:** Daniel Rayson, Sonal Gandhi, Anil A. Joy, Christine Brezden-Masley, Karen A. Gelmon, Sandeep Sehdev, David Cescon, Stephen Chia

**Affiliations:** 1 Division of Medical Oncology, Department of Medicine, Queen Elizabeth II Health Sciences Center, Halifax, NS B3H 2Y9, Canada; 2 Division of Medical Oncology/Hematology, Odette Cancer Centre Sunnybrook Health Sciences Centre, Toronto, ON M4N 3M5, Canada; 3 Division of Medical Oncology, Cross Cancer Institute, Edmonton, AB T6G 1Z2, Canada; 4 Division of Medical Oncology, Mount Sinai Hospital, Toronto, ON M5G 1X5, Canada; 5 Division of Medical Oncology, British Columbia Cancer Centre, Vancouver, BC V5Z 4E6, Canada; 6 Department of Medical Oncology, The Ottawa Hospital Cancer Centre, Ottawa, ON K1H 8L6, Canada; 7 Department of Medical Oncology, University Health Network, Toronto, ON M5G 1Z5, Canada

We appreciate the opportunity to respond to the comment [1] submitted by CADTH in regard to our commentary entitled “Access to Neoadjuvant Pertuzumab for HER2 Positive Breast Cancer in Canada: A Dilemma Increasingly Difficult to Explain” [2] published on 16 December 2022.

The authors are well aware of the important role of CADTH in providing a transparent HTA framework for the assessment of data submitted by industry sponsors. Utilizing clinical and economic perspectives, the process aims to provide an informed recommendation to public and provincial payers regarding the funding of a novel drug (or drug indication) or medical device. The CADTH HTA framework is a critical pan-Canadian process, especially for rapidly evolving and costly novel cancer therapies, with a parallel process in Quebec under INNESS. The CADTH process is supported by all co-authors, with the majority of us having participated over the years as clinical panel chairs or guidance panel members, as well as at higher administrative levels within CADTH and, previously, pCODR.

Most of the comment summarizes the overall CADTH framework, which is readily available on the CADTH website [3]. This is not the main subject of our reply, although we would like to address one issue. We believe it is technically correct that ‘CADTH does not make funding decisions; rather, each public drug plan makes its own decisions based on CADTH’s recommendations.” However, the final CADTH recommendations are directly tied to provincial funding decisions for drugs with national impact. The final recommendation is forwarded to the pan-Canadian Pharmaceutical Alliance (pCPA), which has as its mandate to increase access to clinically relevant and cost-effective treatments as well as achieve consistent and lower drug costs [4]. The pCPA achieves these goals by wielding the combined negotiating powers of the public drug plans from all provinces and territories, as well as the federal government. Our understanding is that a CADTH ‘do not reimburse recommendation’ directly leads to a ‘no negotiation letter’ from the pCPA to the relevant manufacturer, stating that the provinces and pCPA will not enter pricing negotiations. Due to the high cost of most novel cancer therapies, this in turn hinders public funding for therapies with a “Do Not Reimburse” recommendation from CADTH. Thus, although it is technically correct to state that CADTH does not make funding decisions itself, in reality, the final CADTH decision (and INNESS in Quebec) is the essential final element adjudicating funding and public access to most novel cancer therapies in Canada. We have confirmed with the pCPA that there are thus far no provinces that have publicly funded novel cancer therapies with a “Do Not Reimburse” recommendation, given the pivotal weight CADTH has in the process [5].

The comment addresses the specific issue of pertuzumab in the last two paragraphs. Here we would like to highlight our views on the importance of pathologic complete response (pCR) as a clinical endpoint for neoadjuvant therapy as it relates to breast cancer. As discussed in our manuscript, pCR has been accepted as a clinically relevant endpoint at both the individual and trial level by international organizations such as ESMO, NICE, and ASCO. Some of these influential organizations also have robust health technology assessment (HTA) frameworks embedded within their associated health care settings [6]. We also highlight that in Canada, INNESS, as an independent HTA body in Quebec, evaluated the same data and recommended the reimbursement of pertuzumab in the neoadjuvant setting. 

The recognition of the prognostic relevance of pCR in HER2+ breast cancer was gleaned from long-term follow-up of neoadjuvant clinical trials as well as real-world evidence, with consistent observations of significantly worse event-free survival for patients with HER2+ disease that do not achieve a pCR after neoadjuvant therapy. Although the magnitude of improvement in pCR rate required to detect a significant improvement in overall survival outcomes across a trial population is indeed uncertain, there is no uncertainty at the individual patient level. Non-pCR independently and consistently leads to worse survival outcomes for HER2+ and triple-negative breast cancer phenotypes. This observation led to the design of the KATHERINE trial, which tested trastuzumab emtansine as adjuvant therapy specifically for those patients not achieving pCR after neoadjuvant HER2-based therapy and demonstrated significant improvements in disease-free survival (DFS) for this high-risk patient population [7]. This additional adjuvant therapy was recommended for reimbursement by CADTH based on a significant improvement in DFS alone. It is thus clear that the CADTH process can value important endpoints other than overall survival (OS) in the curative setting, and we strongly believe that pCR is one such endpoint.

Although not specifically submitted by the industry sponsor, the incremental improvement in pCR rate afforded by the addition of neoadjuvant pertuzumab would allow treatment de-escalation for approximately one out of six patients. Those additional patients achieving a pCR due to the addition of neoadjuvant pertuzumab would not need 12–14 cycles of trastuzumab emtansine. By rough calculation, we found that this would actually result in cost savings for the public system. Importantly, this would also benefit those patients through the avoidance of toxicities and the additional monitoring required with prolonged adjuvant treatment. This would secondarily translate into better quality of life and likely a faster return to full societal function for individual patients, benefiting the system as a whole. 

Public national health care systems have unique challenges that result in the need for tailored economic models aligned with transparent metrics, priorities, and costs. We are aware that pharmacoeconomic modeling alone does not form the basis for a “do not reimburse” decision by CADTH, but, as expected in a public payer system, this is an important element in the final analysis, and robust pharmacoeconomic data is an essential principle of HTAs [8]. 

CADTH relies on pharmacoeconomic models submitted by sponsors, along with sponsor-submitted data, to develop their own models. In this case, in addition to considering pCR and event-free survival as important endpoints, a cost-savings analysis may have supported a different recommendation. As this approach was not submitted by the sponsor for neoadjuvant pertuzumab, it was not integrated into the CADTH HTA. We believe that a variety of additional modeling methodologies, considering perspectives beyond those submitted by industry sponsors, could be undertaken for cancer drug HTAs with transparency, rigor, and reproducibility. Although in the CADTH process there are opportunities for iterative feedback aimed at clarification and adjustment of relevant parameters from various stakeholders, it appears that the data submitted by the sponsor carries the greatest weight in the analysis. Ultimately, industry sponsors alone may not have the ability or expertise to submit data or models fully capturing important aspects of novel therapies such as neoadjuvant pertuzumab, particularly as they pertain to a single-payer national health care system. 

At the end of the comment, it is stated that ‘a recommendation to not reimburse a drug is issued when the clinical benefit, which is at least comparable relative to other treatments reimbursed by public drug plans at the time of the review, has not been demonstrated.’ Neoadjuvant pertuzumab added to trastuzumab and chemotherapy resulted in a 16.8% improvement in the pCR rate compared to trastuzumab and chemotherapy alone in the relevant trial. At 5 years of median follow-up time, this translated into a 5% absolute improvement in disease-free survival [9]. In combination with treatment de-escalation in the adjuvant setting and potential cost savings, we believe that neoadjuvant pertuzumab demonstrates clearly superior clinical and public health care system benefits compared to the current standard of care. This conclusion was not reached by CADTH, and we believe this demonstrates potential gaps in the alignment of the CADTH HTA with the real-world needs of patients for certain novel therapies. We believe the process should be open to evolving clinical trial endpoints, such as pCR, when they correlate with clinically meaningful improvements in patient outcomes and are supported by both clinical trial and real-world evidence, along with high-level treatment guidelines. 

As clinicians and researchers treating patients with early-stage HER2+ breast cancer on a daily basis, our imperative is to offer relevant curative-intent systemic therapies to the individual patients under our care. The authors are broadly supportive of CADTH and the important work it does in the rapidly evolving cancer treatment landscape, as well as our role as gatekeepers to finite resources within a publicly funded system. However, we believe this case opens an opportunity to re-evaluate the manner in which data is submitted, reviewed, and evaluated using this important Canadian HTA framework. By relying so heavily on industry sponsor-driven data submissions, important additional considerations (e.g., novel clinically relevant endpoints and alternative pharmacoeconomic analyses) may be missed.

As other high-income nations and ongoing clinical trials integrate neoadjuvant pertuzumab as a standard of care for curative-intent treatment of HER2-positive breast cancer, the majority of Canadian patients outside of Quebec will continue to be at a significant disadvantage due to the negative reimbursement decision by CADTH, which closes the door to access.
